# Helicobacter pylori is Associated with Less Fistulizing, Stricturing, and Active Colitis in Crohn’s Disease Patients

**DOI:** 10.7759/cureus.6226

**Published:** 2019-11-25

**Authors:** Andre Fialho, Andrea Fialho, Ammar Nassri, Valery Muenyi, Miguel Malespin, Bo Shen, Silvio W De Melo

**Affiliations:** 1 Internal Medicine: Gastroenterology, University of Florida College of Medicine, Jacksonville, USA; 2 Internal Medicine, University of Florida College of Medicine, Jacksonville, USA; 3 Internal Medicine: Gastroenterology, Tampa General Hospital, Tampa, USA; 4 Internal Medicine: Gastroenterology, Cleveland Clinic, Cleveland, USA; 5 Gastroenterology and Hepatology, Oregon Health and Science University, Portland, USA

**Keywords:** gastroenterology, inflammatory bowel disease (ibd), helicobacter pylori infection, crohns disease, fistulizing crohn’s disease, autoimmune

## Abstract

Introduction

A potential protective role of *Helicobacter pylori* (HP) infection against the development of Crohn’s disease (CD) has been postulated. There is a lack of studies evaluating the association of HP with CD phenotypes. The aim of this study was to investigate the clinical features and disease activity of patients with CD who were diagnosed with HP infection.

Methods

The charts of 306 consecutive patients from the inflammatory bowel disease (IBD) database at the University of Florida College of Medicine, Jacksonville from January 2014 to July 2016 were reviewed. Ninety-one CD patients who were tested for HP were included, and the frequencies of strictures, fistulas, and colitis in surveillance biopsies in these patients were evaluated.

Results

Of the 91 CD patients tested for HP, 19 had HP infection. A total of 44 patients had fistulizing/stricturing disease, and 62 patients had active colitis. In the univariate analysis, patients with HP infection had less fistulizing/stricturing disease (21.1% vs. 55.6%, *p *= 0.009) and less active colitis (42.1% vs. 77.1%, *p *= 0.005). In the multivariate analysis, HP infection remained as a protective factor for fistulizing/stricturing disease phenotype (OR: 0.22; 95%CI: 0.06-0.97; *p *= 0.022) and active colitis (OR: 0.186; 95%CI: 0.05-0.65; *p *= 0.010).

Conclusion

HP infection was independently associated with less fistulizing/stricturing disease and less active colitis in CD patients. Our study suggests CD patients with a history of HP infection are less prone to complications.

## Introduction

*Helicobacter pylori* (HP) is a Gram-negative bacterium that colonizes the gastric epithelium and is the most common bacterial infection worldwide, infecting approximately half of the world’s population [1]. A higher prevalence of HP infection is seen in developing countries with most of the infections being acquired during childhood and persisting throughout adulthood [2]. HP is associated with the development of chronic gastritis, peptic ulcer disease, gastric cancer and mucosa-associated lymphoid tissue lymphoma [3]. In addition, the association of HP infection with extra-gastric disease has been suggested by several researchers [4-12].

Crohn’s disease (CD) is characterized by transmural inflammation of the digestive system and may affect the gastrointestinal tract from mouth to the perianal area. The prevalence is more significant in developed countries with higher latitudes, with prevalence ranging from 10 to 200 per 100,000 people [13]. Complications such as fistulas, perforation, and strictures develop in up to 53% of patients at 10 years follow-up and are associated with significant morbidity [14-15]. Up to 50% of patients will require surgical intervention over the first five years of disease, and many CD patients require multiple abdominal surgeries over their lifetime [16].

The diagnosis of HP infection is associated with a lower incidence and prevalence of CD [17-20]. HP infection is thought to have a protective effect on the development of CD that persists despite eradication treatment and is thought to be secondary to the association of chronic HP infection with the immune system and microbiome modulation [17]. There is a lack of studies in the literature evaluating HP infection in patients diagnosed with CD. The aim of this study was to investigate the clinical features and disease activity of patients with CD who were diagnosed with HP infection.

## Materials and methods

A total of 306 consecutive charts of patients from the inflammatory bowel disease (IBD) database at the University of Florida College of Medicine, Jacksonville from January 2014 to July 2016 were reviewed. Ninety-one CD patients who were tested for HP infection by standard gastric biopsies were included in this study. Detailed clinical data pertaining to HP infection and CD were obtained in this retrospective case-control study. The study was approved by the Institutional Review Board of the University of Florida College of Medicine, Jacksonville and was conducted in accordance with principles in the Declaration of Helsinki.

Inclusion and exclusion criteria

Inclusion criteria were patients with (1) biopsy-proven CD who had a surveillance colonoscopy and (2) esophagogastroduodenoscopy (EGD) gastric biopsies for evaluation of HP. Patients with ulcerative colitis were excluded. Patients without surveillance colonoscopy reports available in our electronic record were also excluded from this study.

Study and control groups

The study group consisted of patients with CD and history of HP, and the control group was patients with CD without HP infection.

Endoscopic diagnosis

The diagnosis of HP was confirmed by gastric biopsies that were fixed in 10% formalin, embedded in paraffin wax, which had 5-mm sections stained with hematoxylin and eosin for histology and with Giemsa staining to evaluate HP status. Random biopsies were collected throughout the colon for the purpose of evaluating CD activity. Those lesions that displayed evidence of dysplasia were followed by surveillance colonoscopies.

Study variables and outcome measurements

A total of 21 variables were studied. Clinical variables included were age, male sex, age at CD diagnosis, disease extension, presence of strictures, presence of fistulizing disease, active colitis on colonics biopsies, symptoms of diarrhea on CD diagnosis, symptoms of bleeding on CD diagnosis, symptoms of abdominal pain on CD diagnosis, history of *Clostridium difficile* infection, arthritis, use of 5-aminosalicylic acid (5-ASA), use of 6-mercaptopurine (6-MP)/azathioprine, use of methotrexate, use of steroids, use of biologics, C-reactive protein (CRP), erythrocyte sedimentation rate (ESR), and stool calprotectin levels. The primary outcomes were to investigate the clinical features and disease activity of patients with CD who were diagnosed with HP infection.

Statistical analysis

Continuous variables were presented as mean ± standard deviation (SD) or N%. Univariate analysis was performed to identify potential variables associated with HP infection in CD patients. Student *t*-tests or nonparametric Wilcoxon rank sum tests were used for continuous factors, and Pearson chi-square tests were used for categorical variables. Multivariate logistic regression analysis was performed to assess the risk factors associated with disease activity in CD patients with HP. An automated stepwise variable selection method performed on 1,000 bootstrap samples was used to choose the final multivariable model. A *P*-value of 0.05 was considered statistically significant. All statistical analyses were performed using SPSS software version 22 (SPSS Statistics for Windows, Version 22.0. Armonk, NY: IBM Corp. 2013.)

## Results

The medical records of 306 consecutive patients with biopsy-proven IBD were reviewed. Of these, a total of 91 CD patients who were tested for HP through standard gastric biopsies were evaluated in this study. Patients were subdivided into HP positive (*n* = 19) or *HP *negative (*n* = 72). The median interval of CD duration was 10 years (interquartile range [IQR] of five to 22 years).

There were no differences between HP-positive and HP-negative patients with regard to age (46.5 ± 3.4 vs. 47.0 ± 1.8; *P *= 0.896), male sex (26.3% vs. 30.6%; *P *= 0.789), age at CD diagnosis (32.3 ± 2.9 vs. 33.7 ± 1.7; *P *= 0.709), disease extension (ileal 10.5% vs. 11.1%; ileocolonic 47.3% vs. 61.1%; colonic 42.2% vs 27.8%; *P *= 0.555), diarrhea on CD diagnosis (78.9% vs. 66.7%; *P *= 0.406), bleeding on CD diagnosis (26.3% vs. 25.0%; *P *= 0.560), abdominal pain on CD diagnosis (89.5% vs 72.2%; *P *= 0.147), history of *Clostridium difficile* infection (17.6% vs. 17.9%; *P *= 0.645), arthritis (10.5% vs. 16.7%; *P *= 0.726), use of 5-ASA (63.2% vs. 70.8%; *P *= 0.580), use of 6-MP/azathioprine (26.3% vs. 29.2%; *P *= 1.000), use of methotrexate (5.6% vs. 4.3%; *P *= 1.000), use of steroids (73.7% vs. 88.9%; *P *= 0.135), use of biologics (36.8% vs. 51.4%; *P *= 0.309), CRP level (5.7 ± 1.8 vs. 23.7 ± 5.3; *P *= 0.112), ESR level (25.0 ± 4.1 vs. 41.1 ± 4.3; *P *= 0.053) and level of calprotectin (187.5 ± 41.7 vs. 159.8 ± 42.5; *P *= 0.727; Table 1)

**Table 1:** Univariate analysis of risk factors associated with H. pylori AZA, azathioprine; CD, Crohn’s disease; CRP, C-reactive protein; ESR, erythrocyte sedimentation rate; 5-ASA, 5-aminosalicylic acid; 6-MP, 6-mercaptopurine *H.pylori positive (12) were tested for *C. difficile*; *H.pylori* negative (56) were tested for *C. difficile.*

Variable	H. pylori positive (n = 19)	H. pylori negative (n = 72)	P-value
Mean age, years	46.5 ± 3.4	47.0 ± 1.8	0.896
Male sex	5 (26.3%)	22 (30.6%)	0.786
Age at CD diagnosis	32.3 ± 2.9	33.7 ± 1.7	0.709
Disease extension			
Ileal	2 (10.5%)	8 (11.1%)	0.555
Ileocolonic	9 (47.3%)	44 (61.1%)	
Colonic	8 (42.2%)	20 (27.8%)	
Diarrhea on CD diagnosis	15 (78.9%)	48 (66.7%)	0.406
Bleeding on CD diagnosis	5 (26.3%)	18 (25.0%)	0.560
Abdominal pain on CD diagnosis	17 (89.5%)	52 (72.2%)	0.147
History of C. difficile infection*	2 (17.6%)	10 (17.9%)	0.645
Arthritis	2 (10.5%)	12 (16.7%)	0.726
Fistulizing/stricturing disease	4 (21.1%)	40 (55.6%)	0.009
Active colitis on random biopsies	8 (42.1%)	54 (77.1%)	0.005
Use of 5-ASA	12 (63.2%)	51 (70.8%)	0.580
Use of 6-MP/AZA	5 (26.3%)	21 (29.2%)	1.000
Use of methotrexate	1 (5.6%)	03 (4.3%)	1.000
Use of steroids	14 (73.7%)	64 (88.9%)	0.135
Use of any biologic	7 (36.8%)	37 (51.4%)	0.309
CRP	5.7 ± 1.8	23.7 ± 5.3	0.112
ESR	25.0 ± 4.1	41.1 ± 4.3	0.053
Fecal Calprotectin	187.5 ± 41.7	159.8 ± 42.5	0.727

A total of 44 patients had fistulizing/stricturing disease, and 62 patients had active colitis identified on random colonic biopsies. In the univariate analysis, patients with HP infection had less fistulizing/stricturing disease (21.1% vs. 55.6%, *p *= 0.009) and less active colitis (42.1% vs. 77.1%, *p *= 0.005) on random colonic biopsies (42.1% vs. 77.1%, *p *= 0.005).

In the multivariate logistic regression analysis, HP, fistulizing/stricturing disease, abdominal pain on CD diagnosis, and active colitis on random biopsies were included in the final model. After adjusting for all other variables in the model, HP infection remained as a protective factor against fistulizing/stricturing disease (OR: 0.22; 95%CI: 0.06-0.97; *p *= 0.022) and active colitis on biopsies (OR: 0.186; 95%CI: 0.05-0.65; *p *= 0.010; Table 2).

**Table 2:** Multivariate analysis of risk factors associated with H. pylori CD, Crohn's disease

Variable	Adjustable OR	95% CI	p
Fistulizing/stricturing disease	0.221	0.06-0.81	0.022
Abdominal pain on CD diagnosis	4.420	0.80-24.34	0.088
Use of steroids	0.242	0.05-1.07	0.061
Active colitis on random biopsies	0.186	0.05-0.65	0.010

## Discussion

HP and CD are two separate disease entities that have a profound impact on the health of affected patients [14,21]. In the present study, HP infection in patients with CD was associated with lower rates of combined fistula or strictures as well as lower rates of active colitis in surveillance colonoscopy with random biopsies. HP infection is acquired in childhood, and it generally persists for life unless treated with antibiotics [22]. HP is commonly associated with chronic gastritis, peptic ulcer disease, and gastric cancer [3]. In addition, HP has been associated with the development of extra-gastric disorders including chronic idiopathic thrombocytopenic purpura and growth retardation in children [5,23]. Strong evidence supports that HP may also be associated with a variety of other diseases such as rosacea, chronic idiopathic urticaria, hyperemesis gravidarum in pregnancy, worsening microalbuminuria in diabetics, ischemic stroke, coronary heart disease, and Grave’s disease [6-11,24]. An inverse relationship with the development of allergies has also been reported [12,25].

CD affects the gastrointestinal tract and is characterized by segmental and transmural inflammation with associated complications ranging from mucosal ulcerative lesions, thickening of the bowel wall, edema of the wall, fistulas and inflammatory or fibrotic strictures with significant morbidity to patients. Given the higher prevalence of CD in developed countries, improved sanitary conditions are thought to reduce exposure to bacterial antigens which protect from auto-inflammation and development of inflammatory bowel disease [17,26]. There is still much to understand the factors affecting disease phenotype and natural history of the disease.

Several studies have shown that people infected with HP had a lower prevalence of CD suggesting a possible protective effect of HP infection against the development of CD [18-20,26]. Lars et al. found that more patients are diagnosed with CD among those who are HP negative than among those HP positive with the protective effect of HP infection persisting even after eradication [17]. In our study, HP infection in patients with CD is associated with lower rates of combined fistulas or strictures and lower rates of active colitis in surveillance colonoscopy with random biopsies. It has been shown that certain intestinal microbial ecosystems harbor aggressive traits relevant in the induction of chronic inflammation in susceptible hosts that can theoretically interfere with CD phenotype [27]. HP infection may interfere with the natural history of CD through modulation of the gastric and intestinal microbiome [28]. Although Th1 responses which are induced by HP favor autoimmune and inflammatory diseases, a potential mechanism by which HP protects against CD has been suggested as HP induces the development of FoxP3+ regulatory T cells and impairs dendritic cell maturation with decreased intestinal inflammation [29-30]. We postulate that this finding is secondary to a potential role of chronic infection with HP to downgrade the auto-inflammatory response seen in CD, potentially changing the intestinal microbiome related to CD development. 

There are limitations to our study. The number of patients with CD that had HP in this study was small, which may have compromised the power of the study. In addition, there may have been selection and referral bias since the study was conducted at a single referral center. Similarly, patients in our study may have had more severe disease and may not be representative of those seen in the general community. Because this was a retrospective study, it was not possible to evaluate other risk factors for such as genetic and inflammatory cytokines associated with different CD phenotypes. It would be interesting to confirm a cause-and-effect association between HP infection, different CD phenotypes and active colitis in CD by future prospective studies.

## Conclusions

In conclusion, HP infection is associated with less fistulizing/stricturing activity in CD and less active colitis on surveillance biopsies. Further prospective studies are needed to confirm this association.

## References

[1] Polk DB, Peek RM Jr (2010). Helicobacter pylori: gastric cancer and beyond. Nat Rev Cancer.

[2] Brown LM (2000). Helicobacter pylori: epidemiology and routes of transmission. Epidemiol Rev.

[3] Malfertheiner P, Megraud F, O'Morain CA (2012). Management of Helicobacter pylori infection--the Maastricht IV/ Florence Consensus Report. Gut.

[4] Mbulaiteye SM, Hisada M, El-Omar EM (2009). Helicobacter pylori associated global gastric cancer burden. Front Biosci.

[5] Fialho AM, Braga AB, Queiroz DM, Rodrigues MN, Herbster ID, Braga LL (2007). The association between Helicobacter pylori infection and height in children from an urban community in north-east Brazil. Ann Trop Paediatr.

[6] Argenziano G, Donnarumma G, Iovene MR, Arnese P, Baldassarre MA, Baroni A (2003). Incidence of anti-Helicobacter pylori and anti-CagA antibodies in rosacea patients. Int J Dermatol.

[7] Federman DG, Kirsner RS, Moriarty JP, Concato J (2003). The effect of antibiotic therapy for patients infected with Helicobacter pylori who have chronic urticaria. J Am Acad Dermatol.

[8] Mansour GM, Nashaat EH (2011). Role of Helicobacter pylori in the pathogenesis of hyperemesis gravidarum. Arch Gynecol Obstet.

[9] Chung GE, Heo NJ, Park MJ, Chung SJ, Kang HY, Kang SJ (2013). Helicobacter pylori seropositivity in diabetic patients is associated with microalbuminuria. World J Gastroenterol.

[10] Zhang S, Guo Y, Ma Y, Teng Y (2008). Cytotoxin-associated gene-A-seropositive virulent strains of Helicobacter pylori and atherosclerotic diseases: a systematic review. Chin Med J.

[11] Shi WJ, Liu W, Zhou XY, Ye F, Zhang GX (2013). Associations of Helicobacter pylori infection and cytotoxin-associated gene A status with autoimmune thyroid diseases: a meta-analysis. Thyroid.

[12] Zhou X, Wu J, Zhang G (2013). Association between Helicobacter pylori and asthma: a meta-analysis. Eur J Gastroenterol Hepatol.

[13] Loftus EV, Jr. Jr., Silverstein MD, Sandborn WJ, Tremaine WJ, Harmsen WS, Zinsmeister AR (1998). Crohn's disease in Olmsted County, Minnesota, 1940-1993: incidence, prevalence, and survival. Gastroenterology.

[14] Ha FJ, Thong L, Khalil H (2017). Quality of life after intestinal resection in patients with Crohn disease: a systematic review. Dig Surg.

[15] Solberg IC, Vatn MH, Hoie O (2007). Clinical course in Crohn's disease: results of a Norwegian population-based ten-year follow-up study. Clin Gastroenterol Hepatol.

[16] Mekhjian HS, Switz DM, Melnyk CS, Rankin GB, Brooks RK (1979). Clinical features and natural history of Crohn's disease. Gastroenterology.

[17] Bartels LE, Jepsen P, Christensen LA, Gerdes LU, Vilstrup H, Dahlerup JF (2016). Diagnosis of Helicobacter pylori infection is associated with lower prevalence and subsequent incidence of Crohn's disease. J Crohns Colitis.

[18] Zhang S, Zhong B, Chao K (2011). Role of Helicobacter species in Chinese patients with inflammatory bowel disease. J Clin Microbiol.

[19] Sonnenberg A, Genta RM (2012). Low prevalence of Helicobacter pylori infection among patients with inflammatory bowel disease. Aliment Pharmacol Ther.

[20] Ando T, Watanabe O, Ishiguro K (2008). Relationships between Helicobacter pylori infection status, endoscopic, histopathological findings, and cytokine production in the duodenum of Crohn's disease patients. J Gastroenterol Hepatol.

[21] Ford AC, Gurusamy KS, Delaney B, Forman D, Moayyedi P (2016). Eradication therapy for peptic ulcer disease in Helicobacter pylori-positive people. Cochrane Database Syst Rev.

[22] Chiesa C, Pacifico L, Anania C, Poggiogalle E, Chiarelli F, Osborn JF (2010). Helicobacter pylori therapy in children: overview and challenges. Int J Immunopathol Pharmacol.

[23] Asahi A, Nishimoto T, Okazaki Y (2008). Helicobacter pylori eradication shifts monocyte Fcgamma receptor balance toward inhibitory FcgammaRIIB in immune thrombocytopenic purpura patients. J Clin Invest.

[24] Guven MA, Ertas IE, Coskun A, Ciragil P (2011). Serologic and stool antigen assay of Helicobacter pylori infection in hyperemesis gravidarum: which test is useful during early pregnancy?. Taiwan J Obstet Gynecol.

[25] von Arnim U, Wex T, Link A (2016). Helicobacter pylori infection is associated with a reduced risk of developing eosinophilic oesophagitis. Aliment Pharmacol Ther.

[26] Cholapranee A, Ananthakrishnan AN (2016). Environmental hygiene and risk of inflammatory bowel diseases: a systematic review and meta-analysis. Inflamm Bowel Dis.

[27] Butto LF, Haller D (2017). Dysbiosis in Crohn's disease - joint action of stochastic injuries and focal inflammation in the gut. Gut Microbes.

[28] Alarcon T, Llorca L, Perez-Perez G (2017). Impact of the microbiota and gastric disease development by Helicobacter pylori. Curr Top Microbiol Immunol.

[29] Rad R, Brenner L, Bauer S (2006). CD25+/Foxp3+ T cells regulate gastric inflammation and Helicobacter pylori colonization in vivo. Gastroenterology.

[30] Arnold IC, Dehzad N, Reuter S (2011). Helicobacter pylori infection prevents allergic asthma in mouse models through the induction of regulatory T cells. J Clin Invest.

